# Pronounced femur malunion after pathological bone fracture due to a simple bone cyst in the shaft of the femur, treated using Ilizarov fixation: a case report

**DOI:** 10.1186/s13256-018-1710-3

**Published:** 2018-06-21

**Authors:** Toru Nishiwaki, Shinichi Uchikawa, Hiroshi Kusakabe, Atsuhito Seki, Yoshitaka Eguchi, Shinichiro Takayama, Akihito Oya, Masaya Nakamura, Morio Matsumoto, Arihiko Kanaji

**Affiliations:** 1 Department of Orthopedic Surgery, Keio University School of Medicine, 35 Shinanomachi, Shinjuku, Tokyo, 160-8582 Japan; 2 Division of Orthopedics, Department of Surgical Subspecialties, National Children’s Medical Center, National Center for Child Health and Development, Tokyo, Japan; 3 Department of Orthopedic Surgery, Fujita Health University School of Medicine, Banbuntane Houtokukai Hospital, Nagoya, Japan

**Keywords:** Pathological bone fracture, Simple bone cyst, Malunion

## Abstract

**Background:**

Although a simple bone cyst carries the risk of pathological fractures, it rarely causes severe deformity. Here we report a case of severe femoral deformity after multiple pathological fractures due to simple bone cysts, and consider the reason for the progression of malunion despite multiple previous treatments. Finally, we propose a treatment option for malunion correction.

**Case presentation:**

A 9-year, 7-month-old Japanese girl was referred to our facility with obvious deformity of her right femur, caused by multiple simple bone cyst-related pathological fractures. The deformity included bowing of approximately 90° and an internal rotation of 60° in the middle third of the femoral shaft. To correct this deformity, we excised the lesion, thus shortening the femur, then corrected the alignment and applied an Ilizarov fixator to extend the bone. At present, 3 years after surgery, the deformity has not recurred and our patient is living without any limitations in daily activities or regular exercise.

**Conclusions:**

When a long bone is in a prolonged state of deformation, the deformity not only progresses as the bone grows, but the soft tissues remain unbalanced and treatment becomes increasingly difficult. To prevent increasing bone deformity and fragility, the deformity should be corrected as quickly as possible using intramedullary nailing or other fixation techniques. We believe that our shortening-distraction method is effective for the treatment of severe deformity with unbalanced soft tissues.

## Background

A simple bone cyst (SBC) is a tumor-like condition of the bone in which a cavity filled with serous fluid forms in the bone marrow, often during childhood and adolescence. Frequent sites of development include long tubular bones, particularly the humerus and femur. In slightly older age groups, a large proportion of cases develop SBCs in the calcaneus; these cases differ from SBCs occurring in the tubular bones [1, 2]. There are several reports on the potential etiologies of SBC, which include anomalous venous drainage [3], prostaglandin, interleukin-1, proteolytic enzymes, nitrogen monoxide involvement [4], and bone reabsorption instigated by parietal cells [5], but none have been confirmed. SBC moves to the diaphysis as the patient grows and may heal without treatment. Thinning of the cortical bone increases the risk of pathological fractures, and pain caused by these fractures often drives the patient to seek medical care. When adjacent to the epiphyseal plate, SBC is believed to be in the active phase, during which the disease is more active and more likely to recur, while in the latent phase, the SBC is separated from the epiphyseal plate [6].

The purpose of treatment is primarily to prevent pathological fractures and to minimize limitations in daily activities. Several treatment options are available; age, sex, level of activity, and location of the SBC play a role in selecting the most appropriate therapeutic approach. Steroid injections [7–10], autologous bone marrow injections [7], and demineralized bone matrix injections [11, 12] are used as conservative treatments. Effective surgical treatments include: curettage; curettage with bone grafting [10]; curettage with bone substitution [6, 10]; curettage with myoplasty, reduced pressure, and shunt method [3]; intramedullary flexible nails [13–15]; and a combination of these.

The combined use of Ilizarov fixation and bone extension is a treatment method often used for rebuilding in cases of infection, pseudarthrosis, and bone defects after removing bone tumors. Excision of the lesions is another treatment option for SBCs, but in recent years, less invasive treatments have become preferable; thus, it is extremely rare for excision, Ilizarov fixation, and bone extension to be used in combination for the treatment of SBC. In addition, in our extensive literature review, we found no reports of bone extension implemented simultaneously with excision of the lesions and correction of deformations in combination with Ilizarov fixation.

Here we report the use of Ilizarov fixation with bone extension to correct deformations in a patient with SBC of the femur who showed repeated pathological fractures and a clearly progressing deformity, despite multiple previous treatments.

## Case presentation

*Patient*: a 9-year, 7-month-old Japanese girl (height 127 cm, body weight 33 kg, body mass index 20.5 kg/m^2^).

*Primary complaint*: severe deformity of the femur.

*Past medical history*: no notable history.

### History of present illness

**Fig. 1:**
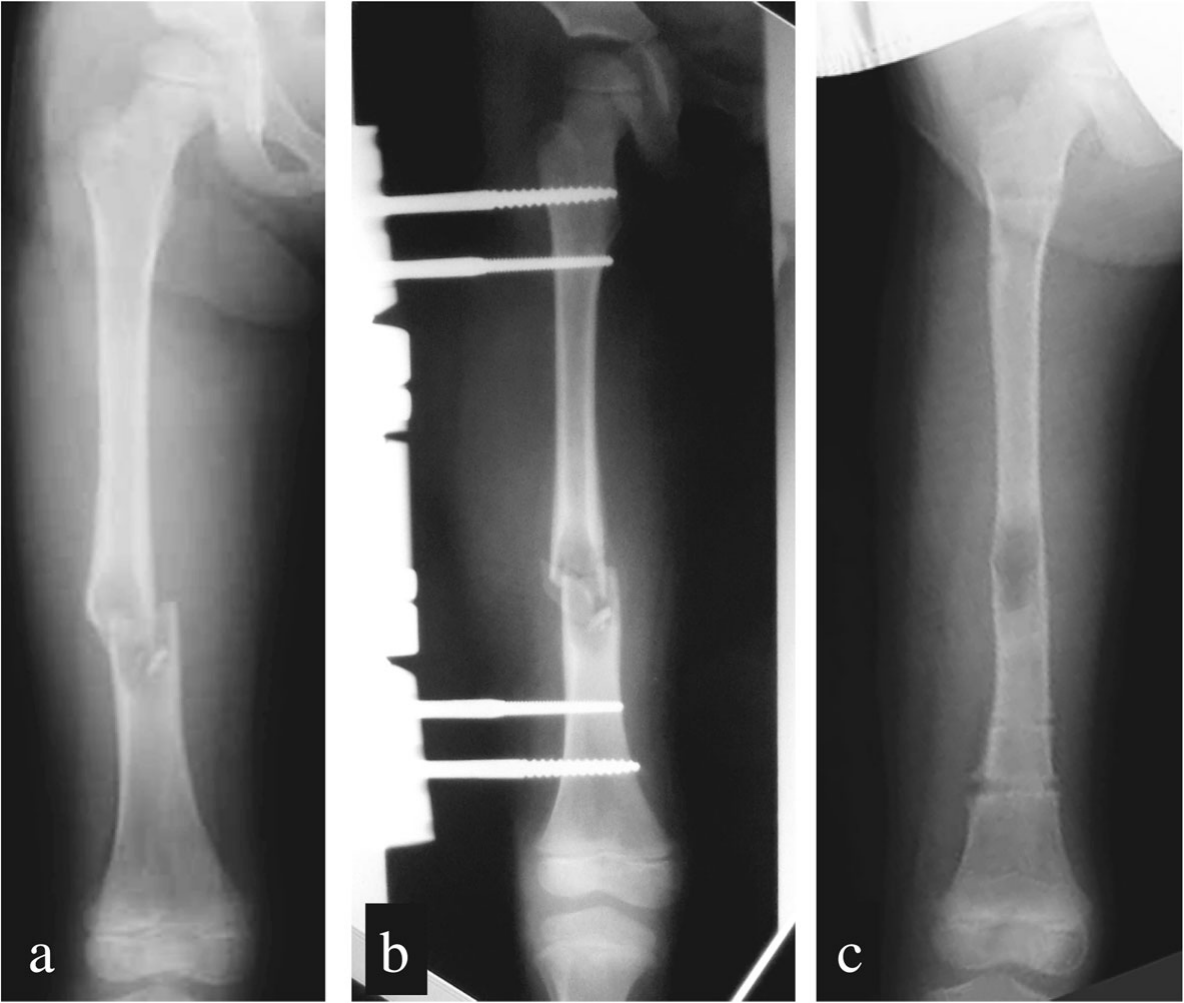
Right femur frontal radiographs. **a** Pathological bone fracture in the right femur at age 4 years, 6 months. **b** After lesion curettage/external fixation; (**c**) bone fusion and removal of the fixator

**Fig. 2:**
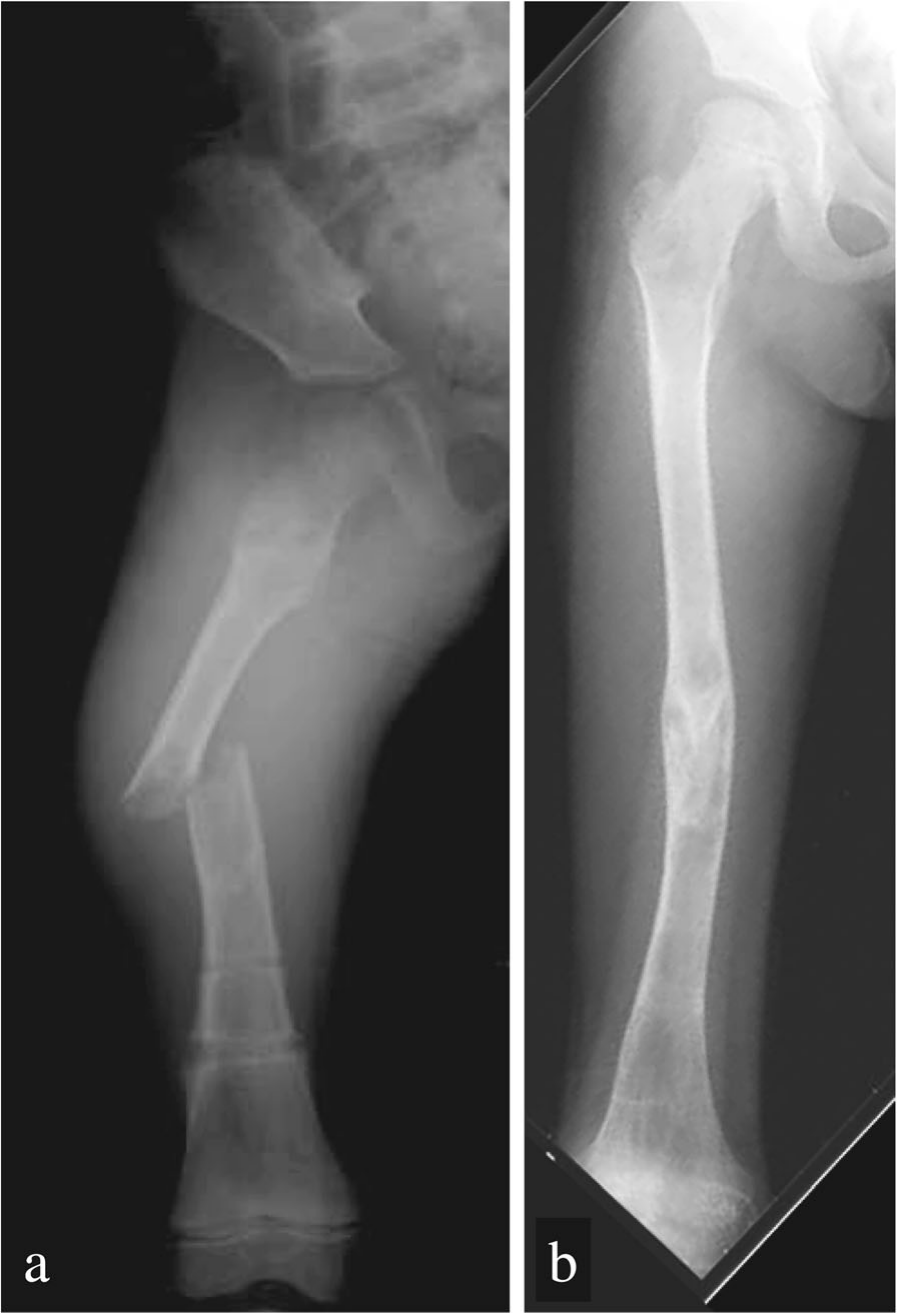
Right femur frontal radiographs. **a** Second fracture, at 1 week after removing the external fixation. **b** At the time, when bone fracture had healed using conservative therapy

The patient was referred to our facility with complaints of progressive deformity of her right femur associated with an SBC and pathological fractures. The girl experienced a pathological fracture of her right femur due to bone tumor when she was 4-years, 6-months old (Fig. 1a), which her previous physician treated with lesion curettage and fixation (Fig. 1b). Pathological findings confirmed the presence of an SBC. Bone healing was confirmed 6 months later, at the age of 5 years. The fixator was removed and steroids were injected simultaneously with an artificial bone graft into the lesion (Fig. 1c). She wore a functional brace after the surgery. However, 1 week after removing the fixator, a new fracture developed in the same location of the bone, following a minor external injury (Fig. 2a). After a 5-week trial of conservative treatment using steel wire skeletal traction, she underwent fixation with application of a hip spica cast. Five months after the second fracture, at the age of 5 years and 5 months, weight-bearing on the affected limb was progressively initiated, and she was discharged with full weight-bearing status at the age of 5 years and 7 months. She was monitored as an out-patient (Fig. 2b).

**Fig. 3:**
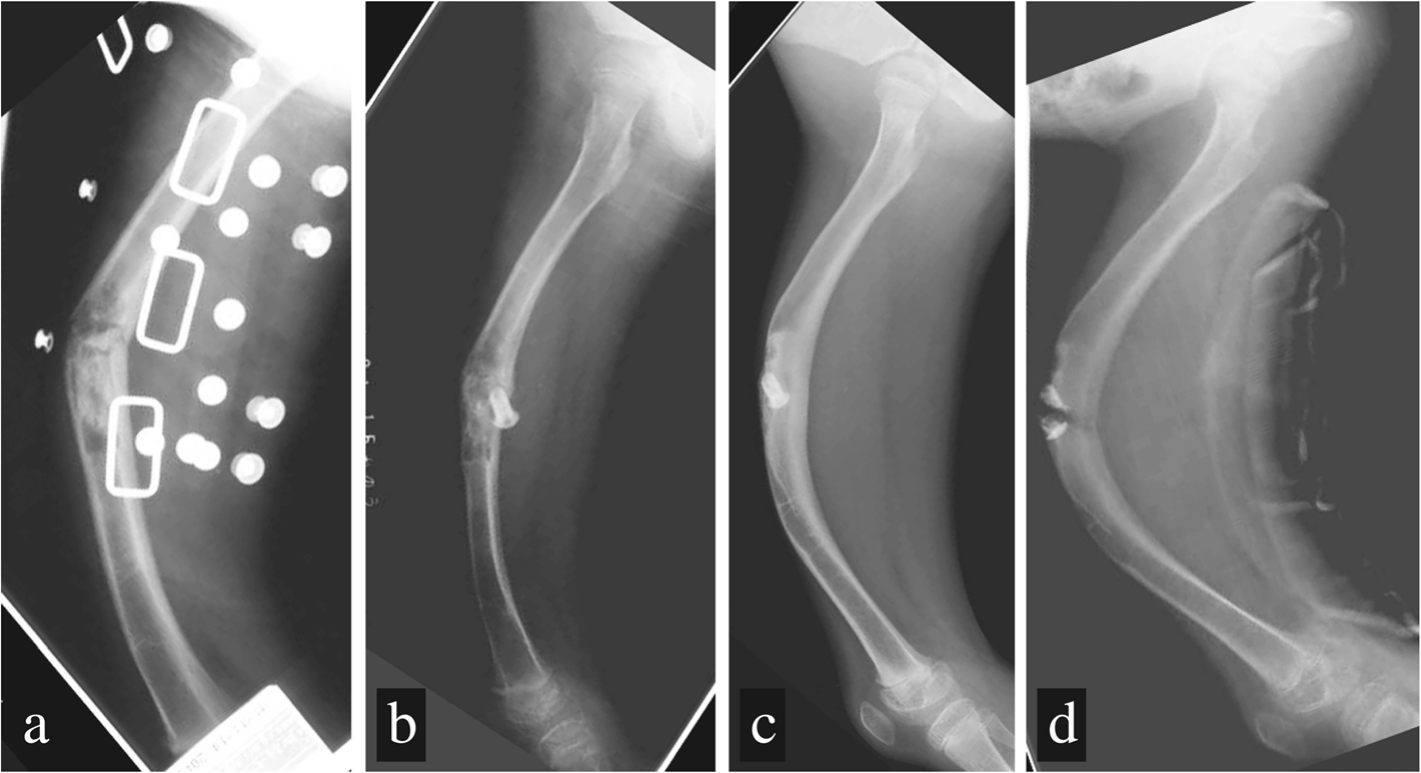
Right femur lateral radiographs. **a** Third fracture at age 6 years, 4 months. **b** After the bone fracture had healed with the cannulated screw inserted. **c** At age 9 years, 3 months, the patient wore a functional brace, but the bowing deformation gradually worsened. **d** At age 9 years, 6 months, femur fracture developed at the cannulated screw

Subsequently, she developed a fracture again at the age of 6 years and 4 months, following a fall (Fig. 3a). She was once again treated with a steel wire skeletal traction. Once bone healing was achieved, the inside of the cyst was curetted and a cannulated screw was inserted to reduce localized pressure (Fig. 3b). Approximately 6 weeks after surgery, a hip spica cast was applied, which was followed by the use of a functional brace. During the follow-up period, severe bowing of her right femur developed, which was progressive (Fig. 3c). At the age of 9 years and 6 months, the femur fractured, with the fracture originating at the cannulated screw. The screw was subsequently removed (Fig. 3d) and the patient was referred to our facility at the age of 9 years and 7 months.

**Fig. 4:**
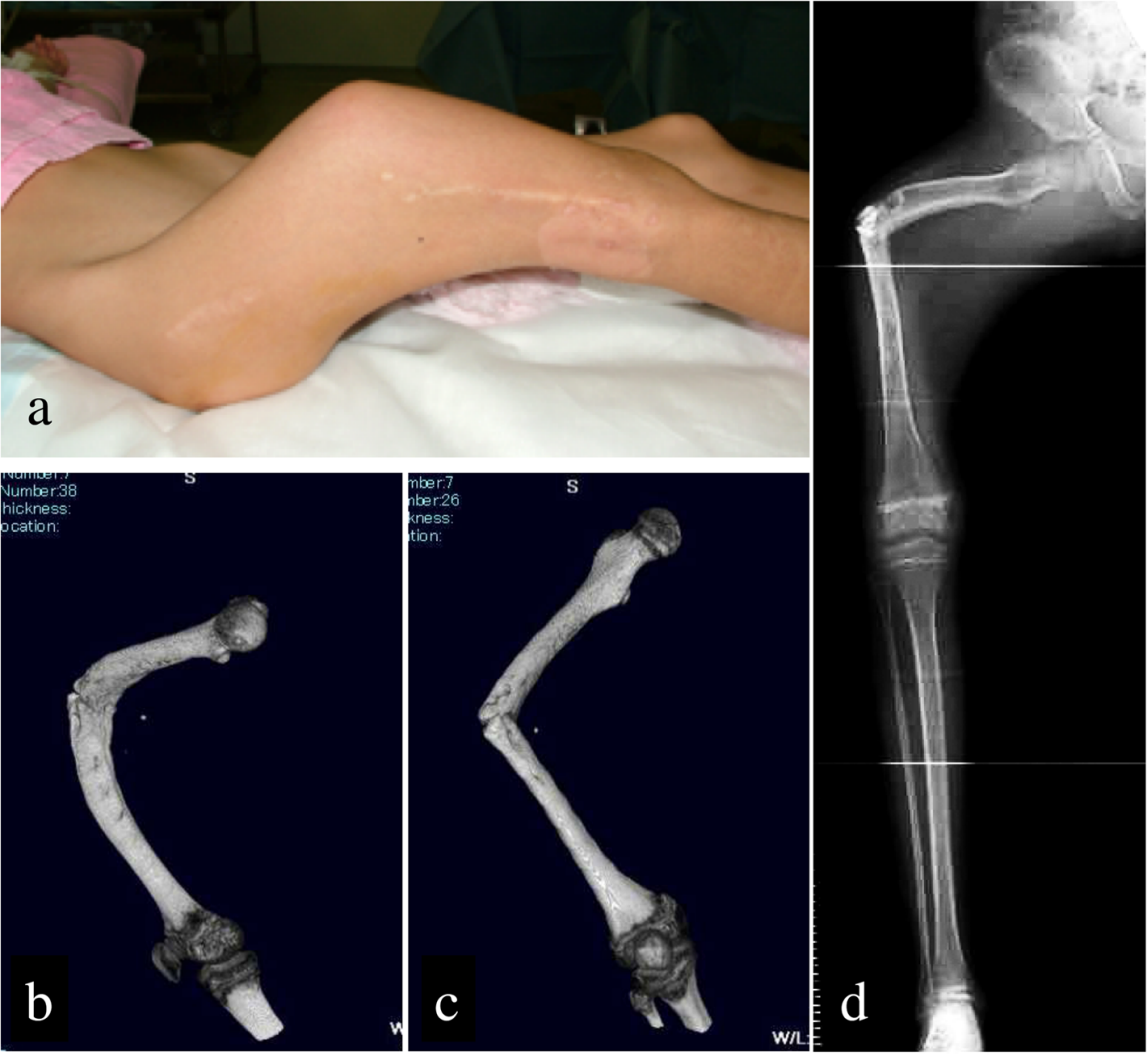
At age 9 years, 7 months upon visiting our facility. **a** External appearance of the right femur. **b**, **c** Right femur three-dimensional computed tomography. **d** Simple radiograph of the entire right lower limb. Upon first examination at our facility, the right femur was deformed, with bowing of approximately 90° and internal rotation of 60° with incomplete fractures in the same area

**Fig. 5:**
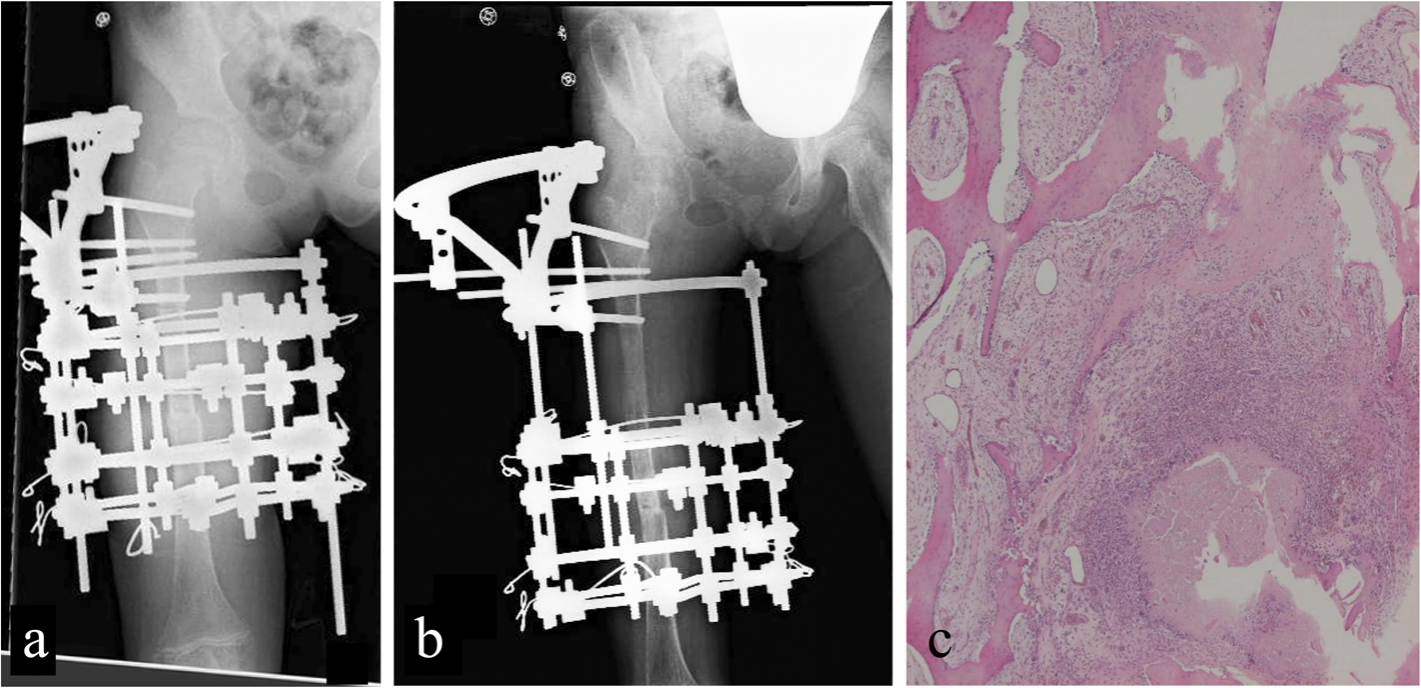
**a** Radiograph immediately after surgery. After extension, the segment of bone with the lesion was removed, shortening and correcting the alignment permanently. **b** Simple radiograph after extension, approximately 3 months after completing extension. **c** Pathological examination of surgically resected specimen (magnification × 100). Histopathology of the extracted lesion shows a small formation considered to be inorganic material scattered in the medullary cavity, consistent with fracture due to bone cyst

On first examination at our facility, the femur was deformed, with a bowing of approximately 90° in the central area of the femur and an internal rotation of 60°, with incomplete fractures observable in the same area (Fig. 4). We proceeded with resection of a 7-cm portion of the bone, which included the SBC, corrected the alignment and applied an Ilizarov fixator to gradually lengthen the thigh. We also cut a portion of healthy bone from the proximal femur (approximately 10 cm from resected lesion), for use as bone extension at the site of resection (Fig. 5a). One week after surgery, extension of the bone was initiated at a speed of 1 mm/day, completing the process in 4 months. Fixation was then maintained until the callus matured (Fig. 5b). Once callus maturation was achieved, the external fixator was gradually removed to prevent re-deformation.

On histopathologic examination of the surgically resected bone, fibrillation of the medullary cavity was increased, and a small formation, identified as inorganic-like material, was scattered throughout; these findings were consistent with a fracture due to a bone cyst (Fig. 5c).

**Fig. 6:**
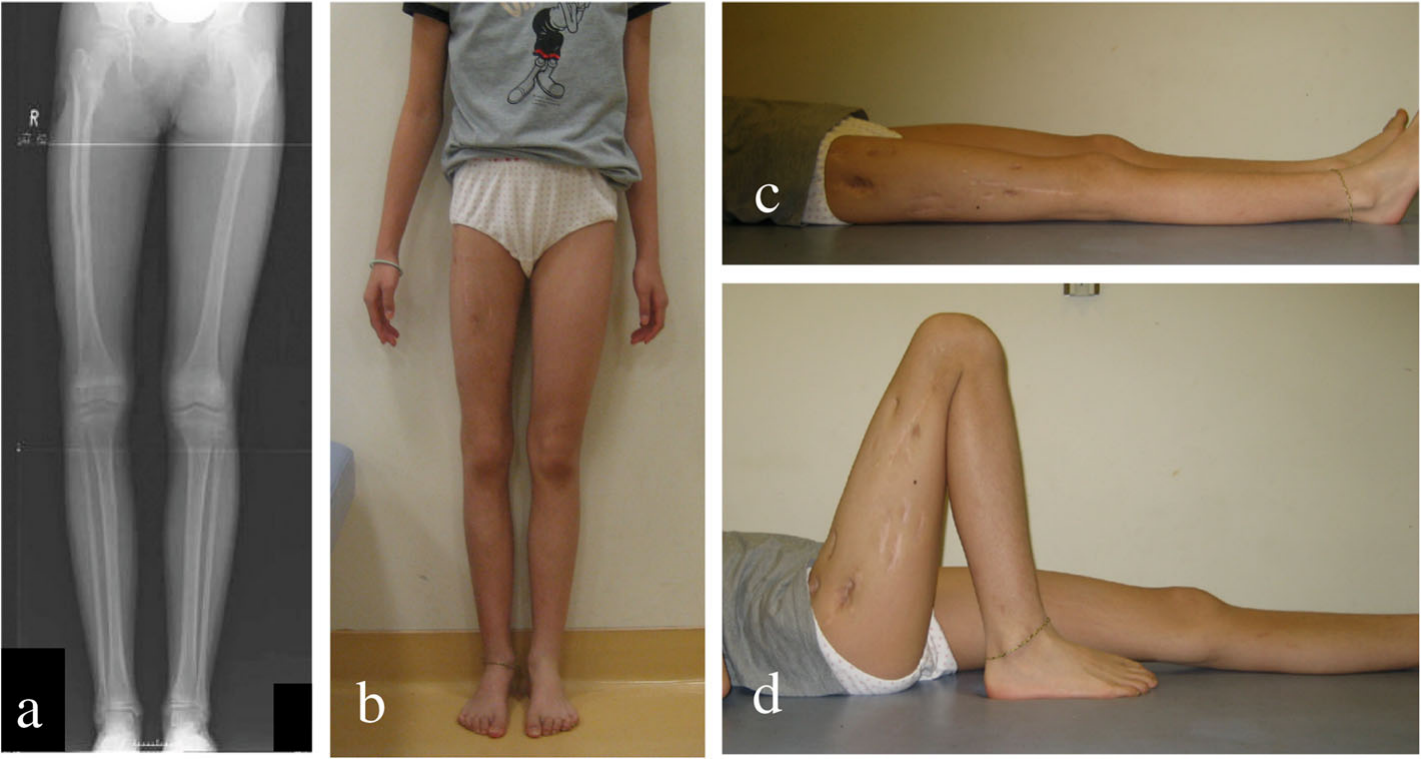
At the age of 12 years, 10 months. **a** Frontal radiograph of both limbs. **b** External appearance of both limbs. **c** External appearance of knee extension. **d** External appearance of knee flexion. Alignment of the right femur was successful and most of the difference in length was corrected

At present, 3 years after surgery, correction of the deformity has been maintained, and our patient does not experience any limitations in daily activities or regular exercise (Fig. 6).

## Discussion and conclusions

There are several reports on the successful treatment of SBC in long bones using: curettage/bone graft (artificial or autologous); infusion therapy, such as steroids and autologous bone marrow liquid; and drilling hollow screws to reduce pressure [3, 7–12]. In our patient, curettage and steroid infusion into the cyst, and reducing pressure using a cannulated screw were previously attempted. After inserting a cannulated screw, the cyst disappeared over a protracted period of time, even though the cyst was in a latent phase. The histopathology of this lesion was interesting as it differed from the normal process but, unfortunately, we were unable to obtain pathological information from our patient’s previous doctor.

Normally, in childhood, autologous corrections are successful, even for the treatment of a deformity of the femur. Malkawi *et al.* reported successful autologous correction of a 30° sagittal plane and 20° coronal plane deformity of the femur in a pediatric patient [16]. In our patient, new bone formation was observable in the affected region of the femur due to sequential autologous correction following the multiple fractures (Fig. 3b, c). However, the deformity progressed, probably due to the fragility of the bone and excessive tension applied by the unbalanced surrounding soft tissues. In such cases, where the bone is fragile and the deformity is progressing, there is a possibility of severe functional disability and, therefore, early and active treatment should be considered, including treatment of the SBC and stabilization of the fracture. The efficacy of intramedullary flexible nailing for the treatment of pathological fractures due SBCs has been reported in several studies [13–15]. In cases of progressive deformity despite adequate treatment, our approach of resection of the lesion, correction of the alignment, and extension of the bone may provide the most effective treatment.

As previously mentioned, excessive tension applied to the bone by the unbalanced surrounding soft tissues was likely to be an important factor contributing to the progression of the bone deformity. In particular in our case, the severity of the deformity hindered lengthening of the soft tissue with bone growth, with the iliotibial tract being particularly tight, which would have applied abnormal excessive tension on the bone, thereby increasing the severity of the deformity. Considering the dynamic effects of unbalanced soft tissues on the deformity, treatment using a correction osteotomy alone would probably have been insufficient to maintain bone alignment and length.

Several techniques have been designed to facilitate rebuilding of bone after resection of a bone tumor, including the use of vascularized autologous bone grafts, an autologous bone graft (Pasteur-treatment, liquid cyst treatment, radiotherapy, radiation irradiation, and others), allogeneic bone graft, bone extension, and other implants. Typically, a stable biological rebuilding method is ideal for young individuals who are still growing. Bone extension is one of these methods and is often used for the treatment of bone deformity and deficiency, such as caused by trauma, osteomyelitis, and pseudarthrosis. Tsuchiya *et al.* reported the efficacy of rebuilding using bone extension after resection of bone tumors in 19 children during their growth period [17]. Specifically, the authors reported on ten cases of bone transport (five cases of osteosarcoma and five of osteoclastoma), three cases of shortening-distraction (two cases of osteosarcoma and one case of Ewing’s sarcoma), and six cases of bone transport with medullary pin (three cases of osteosarcoma, two cases of mild osteosarcoma, and one case of malignant histiocytosis). The average bone loss in these cases was 8.4 cm. On final follow-up, function was assessed as excellent in 12 patients, good in 5 patients, and fair in 2 patients. Complications occurred in ten patients, but the report stated that all cases were ultimately completely cured. In our review of the literature, we did not identify any case reporting the use of our method, namely excision of the lesion, correction of the alignment, and subsequent lengthening of the bone, for the treatment of a deformity caused by a bone tumor. For our patient, we considered the following two potential methods to correct alignment and lengthen the bone after removing the lesion: shortening of the bone (with SBC resection), and correction of alignment and subsequent lengthening of the bone; or soft tissue release, including the iliotibial tract, maintaining the length and alignment of the bone and filling the bone deficiency with vascularized autologous bone graft or bone transport. Considering the invasiveness of soft tissue release and the ease of bone resection with subsequent lengthening, we deemed this latter approach as being the most conducive to achieving favorable clinical and functional outcomes, including gradual lengthening of soft unbalanced soft tissues.

Therefore, for cases of progressive long bone deformity in a young child, associated with unbalanced soft tissues, we believe that our shortening-distraction method can be an extremely effective treatment that can be used to provide early correction and stabilization of the long bone.
